# The Effects of Regional Muscle Strength and Mass on Standing Long Jump Performance

**DOI:** 10.3390/muscles3010007

**Published:** 2024-03-04

**Authors:** Yuki Nakai, Yujiro Usumoto, Yasufumi Takeshita

**Affiliations:** 1 Department of Mechanical Systems Engineering, Faculty of Engineering, Daiichi Institute of Technology, 1-10-2 Kokubuchuo, Kirishima 899-4395, Japan; y-takeshita@daiichi-koudai.ac.jp; 2 Department of Physical Therapy, Kagoshima Daiichi Medical Rehabilitation College, 1-12-42 Kokubuchuo, Kirishima 899-4332, Japan; y-usumoto@tsuzuki-edu.ac.jp

**Keywords:** abdominal pressure, body composition, jumping power, muscle output, standing broad jump

## Abstract

Muscle strength and mass strongly influence performance. The role of the trunk, upper limbs, and lower limbs in a specific performance is important but unclear in terms of muscle strength, muscle mass, and the degree of influence of each part. Standing long jump is a performance that produces results by not only the muscles of the lower limbs working together but also the entire body, including the trunk and upper limbs. To determine the influence of muscle strength and the mass of each body part on standing long jump, 31 healthy young adults (18 males and 13 females) participated in this study. Abdominal trunk muscle strength, grip strength, and knee extension muscle strength were measured, each of which was defined as trunk, upper limb, and lower limb muscle strength. The trunk, upper limb, and lower limb muscle masses were measured using a body composition analyzer. Performance was measured using the standing long jump test (jumping power). Factors influencing standing long jump were examined. A multiple regression analysis revealed that trunk (β = 0.367, *p* = 0.006) and upper limb (β = 0.608, *p* < 0.001) muscle strength values were extracted for standing long jump (adjusted R^2^ = 0.574, *p* < 0.01). Trunk and upper limb muscle strength influence standing long jumps.

## 1. Introduction

Strength training is effective in increasing muscle strength and mass and affects a variety of performances [1]. Generally, a positive correlation has been established between muscle cross-sectional area and maximal muscle strength [2]. This relationship is particularly evident when muscle mass is high [3]. Therefore, the greater the muscle mass, the greater the force generated. Increased muscle mass increases strength and power; hence, weight gain due to increased muscle mass may be advantageous [4]. Although muscle mass is important for athletes, movement speed is also important. Given the relationship between force, mass, and acceleration according to Newton’s second law of motion, performance is greatly influenced by the characteristics of the muscle strength and mass possessed by an individual. The positive relationship between muscle strength and muscle mass is also being questioned [5,6]. Taking these into account, it is important to consider whether muscle strength or muscle mass should be prioritized for specific performance in sports; to the best of our knowledge, no report has examined both.

In strength training, the establishment of target areas is an important factor in achieving individual goals [7]. The relationship between trunk, upper, and lower limb functions and performance in sports has been reported. High trunk muscle strength improves trunk stability, decreases the risk of back injury, and improves athletic performance [8]. Poor trunk stability is associated with the development of lower extremity injuries [9]. Increased trunk muscle endurance is associated with improved shoulder mobility and stability, which are associated with improved performance [10]. In an optimal control simulation of the standing long jump (SLJ), participants with unrestricted arm motion were able to jump 40 cm higher than those with restricted arm motion [11]. Combined upper and lower extremity training has been reported to improve physical function in athletes [12]. Although the relationship between the trunk, upper limbs, and lower limbs and performance has been demonstrated, there are no reports on which parts of the trunk, upper limbs, and lower limbs contribute more to specific performances.

Trunk evaluation can be performed by measuring muscle strength during flexion-extension using a dynamometer [13] and a body composition analyzer [14]. No study has examined the relationship between the trunk, upper limb, and lower limbs and performance, including the evaluation of trunk muscle strength (including the deep trunk muscles) using isometric contraction of the trunk muscles alone. The SLJ is considered a general indicator of the strength and power system performance of young people [15]. The SLJ is also reported to be a low-cost and highly reliable test with few equipment requirements [16]. The purpose of this study was to examine whether the trunk or upper and lower extremity strength or muscle mass contributes more to the SLJ and to determine which parts of the trunk and upper and lower extremities contribute more to the SLJ. The identification of these factors may serve as indicators for the planning and implementation of training programs.

## 2. Results

**Figure 1:**
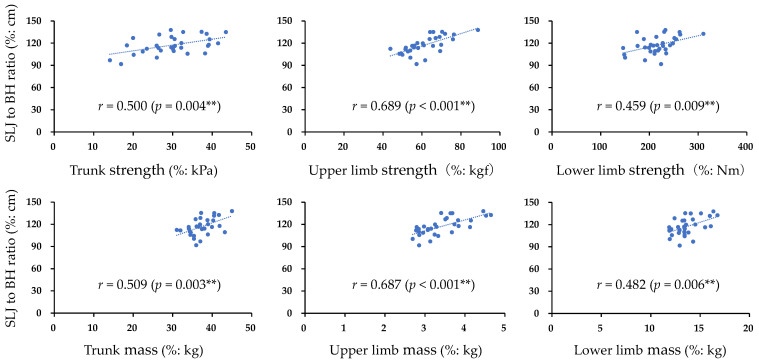
Correlation between normalized items and normalized SLJ: SLJ, standing long jump; BH, body height; *r* correlation coefficient; ** *p* < 0.01.

The participant characteristics and measurement results of each muscle strength, muscle mass, and SLJ are presented in Table 1. Trunk muscle strength showed a high intraclass correlation coefficient (ICC_1,3_) of 0.980 (95% confidence interval (CI) = 0.964–0.990, *p* < 0.001). Furthermore, Figure 1 shows the correlations of trunk, upper limb, and lower limb muscle strength and muscle mass with SLJ. Significant positive correlations were found between the SLJ and all items (*p* < 0.05). Table 2 presents the results of the multiple regression analysis. The significant extracted items affecting the SLJ were trunk muscle strength (β = 0.367, 95% CI = 0.184–0.972, *p* = 0.006) and upper limb muscle strength (β = 0.608, 95% CI = 0.432–1.036, *p* < 0.001) (adjusted R^2^ = 0.574).

## 3. Discussion

This study examined the degree to which the strength and muscle mass of the trunk and the upper and lower extremities affect performance. The dependence of the SLJ distance on the speed and angle of movement of the center of gravity has been previously reported [17]. We need to increase the speed of movement at the center of gravity and keep the hip joint anteriorly tilted during takeoff, while the thighs should be pulled in during landing [18]. Ashby et al. used optimal control simulations to show that arm swinging during SLJ generates greater work in the upper extremity muscles and effectively transfers energy to the lower extremities [11]. Jumps have also been reported to occur by transferring energy to other parts of the body prior during takeoff through the upper extremity swing and recoil, which increases the velocity and displacement of the center of gravity in both the horizontal and vertical directions [19]. When the arms move beyond the horizontal position, the vertical forces on the shoulders become upward forces acting on the trunk [20]. Increased grip strength is positively related to golf swing speed and driving performance, which are linked from the upper extremities to the whole body [21,22]. Considering the findings of previous studies, the present study suggests that upper-extremity muscle strength contributes to a stronger arm swing, influences the center-of-gravity transfer velocity and angle, and contributes significantly to the SLJ.

Movement and force from the upper extremities to the hip joint are linked via the myofascia of the trunk, hip joint, and pelvic area [8,23]. These act as local stabilizers and thus play an important role in maximizing performance [8]. In particular, effective mobilization of the trunk muscles is associated with precise control of the lumbar–pelvic–hip motion and optimal generation of muscle strength [24,25]. Abdominal muscles are activated sequentially from deep to shallow muscles during the SLJ and are most active during the takeoff phase [26]. Studies have shown that muscles that stabilize the trunk are always activated before moving the limb [27]. This is thought to be a function that aids in limb force or power generation during kinetic chain activity [28,29]. It has been suggested that a strong and stable trunk and its rapid activation underlie power generation in the limbs and are beneficial in achieving high sports performance [8]. Thus, in addition to upper extremity strength, trunk muscle strength may contribute to the SLJ.

We investigated whether absolute strength due to muscle mass contributes more to SLJ, which represents jumping power, or the relative strength of an individual who must move his or her own body weight. The contribution of muscle mass to the SLJ was not determined in this study. Previous studies have shown a strong correlation between muscle cross-sectional area and muscle strength [30]. Despite this association, individual differences in the relationship between muscle mass and strength should be noted. Muscle mass is the foundation of muscle structure, muscle fiber type, and exercise unit mobilization and activation [31,32]. Muscle mass may affect performance not only by muscle cross-sectional area but also by muscle fiber diameter [33]. On the other hand, muscle strength can be influenced by physiological and neurological factors other than muscle mass [34]. In experienced athletes, increased muscle strength often leads to improved performance. High performance levels can be achieved by combining muscle strength and task specificity [31,32]. Strength is important in tasks that require moving the body while maintaining velocity [35]. For individuals with athletic experience, the simple task of SLJ, which requires explosive horizontal jumping power, may directly involve muscle strength and may be a disadvantage for muscle mass. Thus, the SLJ results of participants in this study who had previous athletic experience would not have involved muscle mass; however, muscle strength would have contributed directly to performance.

This study has several limitations. First, we included only healthy young adults; therefore, it is unclear whether the results are applicable to other age groups or a variety of competitive athletes. Second, statistical analysis combined male and female performance results. Indeed, sex differences in form and absolute performance are known to exist [36]. However, between the ages of 60 and 80, the decline in jumping performance for both sexes drops to about half that of younger age groups. No sex differences could be recognized in the comparison of normalized jumping parameters in jumping events [37,38]. All normalized values were also used in this study to account for body size, and sex and age were added as adjustment factors in the multiple regression analysis. Third, performance is multidimensional as it involves technique and visual–cognitive factors in addition to muscle function [39]; however, these factors were not examined in this study. Fourth, grip strength was used as a measure of upper limb muscle strength, and knee extension muscle strength was used as a measure of lower limb muscle strength; however, these relationships are not strictly consistent. Fifth, SLJ has been shown to be strongly associated with each of the lower and upper body strength tests [15]. However, lower-limb muscle strength was not selected as a variable for the optimal model in the stepwise method in this study. This does not mean that lower limb muscle strength is unaffected in SLJ. In SLJ, adjustments in movement and technical skills influence the final performance results [18]. The adjustment aspect may have influenced the selection of trunk and upper limb strength as the optimal models in this study. Finally, this study was cross-sectional, but it did not examine long-term effects. Therefore, further longitudinal studies based on these findings are required.

## 4. Materials and Methods

### 4.1. Participants

A power analysis was conducted using G*Power 3.1.9.2 (Kiel University, Kiel, Germany) with a general correlation coefficient r of 0.5, which corresponds to a large effect size classification, and a power of 0.80, alpha 0.05 [40]. This study was found to require at least 29 participants. The recruitment was announced via social networking services to students at one engineering university and one healthcare professional school in Kirishima City. Young adults were recruited and 32 were enrolled, considering a dropout rate of 15%. The participants’ eligibility was assessed using a questionnaire. The exclusion criteria included the following: presence of pain, history of neurological disease, history of musculoskeletal disease, or history of surgery within 1 year. As a result of the criteria, the study participants comprised 31 (18 male and 13 female) individuals, excluding one with a history of surgery, with a mean age, height, and weight of 20.3 ± 1.4 years, 165.7 ± 9.0 cm, and 62.4 ± 12.2 kg, respectively. They played leisure sports for 1.4 ± 1.8 h/week, had 4.7 ± 3.5 years of competitive sports experience, and were not competitive athletes at the time of measurement.

### 4.2. Ethics

This study was approved by the Ethics Committee of the Daiichi Institute of Technology (R4-002). All participants were informed orally and in writing, in accordance with the Declaration of Helsinki. Written informed consent was also obtained from all participants.

### 4.3. Study Design and Procedures

Using a questionnaire, all participants were asked about their age, sex, medical history, dominant hand and leg, exercise history, and exercise habits. The participants’ body height and body composition measurements were taken to measure muscle mass by region, and 5 min of aerobic exercise (Well Bike BE-200 FUKUDA DENSHI, Tokyo, Japan) was performed as a warm-up (70 rpm and 80 W for men and 70 rpm and 50 W for women) [41]. Subsequently, the SLJ was measured for physical performance, and the abdominal trunk muscle strength, grip strength, and knee extension muscle strength were measured to determine muscle strength.

### 4.4. Measurement Methods

Body height was measured using a height meter (seca213, seca, Chiba, Japan) with the participants standing in an upright position with bare feet. Measurements were taken to the nearest ±0.1 cm. Body weight and trunk and upper and lower limb muscle masses were evaluated through bioimpedance analysis (BIA) using a TANITA MC-780MA (MC-780MA, Tanita, Tokyo, Japan). This instrument is an 8-electrode, multi-frequency (5 kHz/50 kHz/250 kHz) body composition analyzer that predicts muscle mass based on resistance and reactance [42]. The validity of this device in healthy young adults has been reported [43]. Body composition analysis was performed for approximately 1 min by holding the two handles with the arms away from the trunk. BIA was performed according to the following standard operating procedures after referring to general recommendations [44]. (1) Using an air conditioner, maintain the room temperature at approximately 25 °C; (2) measurements should be carried out at least 2 h after a meal; (3) at least 2 h after exercise; (4) participants should empty their bladder and bowels before measurement; (5) measurements should be carried out at the same time of the day (between 3:00 p.m. and 5:00 p.m.), taking into account the circadian rhythm of the body; (6) metal objects should be removed and light clothing should be worn; and (7) the weight and height of the participant should be measured to the nearest ±0.1 kg and ±0.1 cm, respectively. Values for the dominant hand and leg were normalized according to body weight.

**Figure 2:**
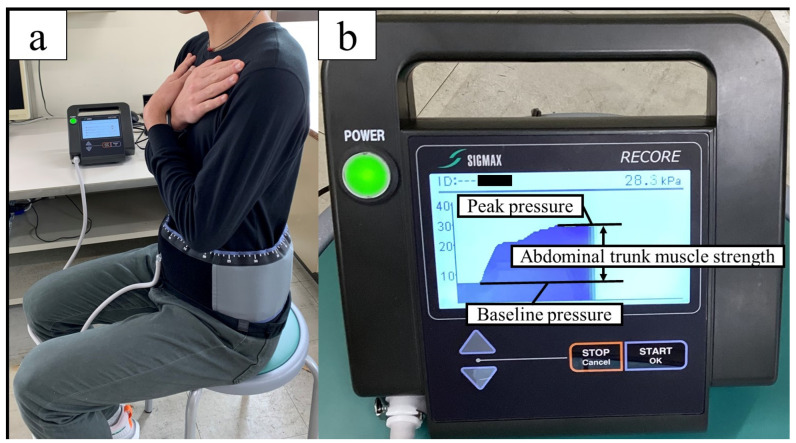
Measurement of abdominal trunk muscle strength (**a**) Wrap a cuff belt around the abdomen and inflate it; (**b**) the difference between the peak pressure and the base pressure is the abdominal pressure generated (abdominal trunk muscle strength).

Abdominal trunk muscle strength was evaluated using an abdominal trunk muscle strength measuring device (RECORE^®^, Sigmax, Tokyo, Japan) that measures abdominal pressure by wrapping a cuff belt around the abdomen (Figure 2a). After applying approximately 5 kPa of pressure to the abdomen (baseline pressure), the participants were instructed to exert maximum abdominal pressure (peak pressure), and the amount of pressure change (peak pressure–baseline pressure) was defined as the abdominal trunk muscle strength (Figure 2b) [45]. This device assessed the muscle activity of the diaphragm, rectus abdominis, internal oblique, external oblique, transverse abdominis, and pelvic floor muscles [46]. This method is similar to the “abdominal bracing” maneuver, which is one of the most effective techniques for increasing trunk stability [47]. Both techniques are used to increase trunk stability through voluntary co-contraction of the abdominal muscles. Maximal isometric strength is the maximum force a person can exert without changing body position. Fixing the body position keeps the muscle length constant and reduces fluctuations caused by changes in joint angles and movement speeds [48,49]. Maximum muscle strength can be demonstrated statically and with a high reproducibility, and the measurement is relatively easy [50]. A previous study using this device and using the same procedure showed ICCs_1,3_ of 0.975–0.983 (95% CI = 0.952–0.992, *p* < 0.001) [45]. The participants practiced once or twice under submaximal force, and after sufficient rest, three measurements were taken, with 1 min intervals between each measurement. The participants were given verbal encouragement while performing the test. The maximum value was recorded as a measure of the trunk muscle strength. Data were normalized by weight.

We previously tested whether this device reflected trunk muscle activity in 13 healthy young adults. EMG signals were collected using a surface electromyograph, Ultium EMG (Noraxon Inc., Scottsdale, AZ, USA), to measure muscle activity while wearing RECORE^®^. The EMG equipment specifications included a frequency response of 10–500 Hz, variable input impedance >100 MΩ, common mode rejection > 100 dB, and a sampling frequency of 2000 Hz. First, each participant’s maximum abdominal trunk muscle strength (100%) was measured three times using RECORE^®^’s measurement mode, and the maximum value was used. The ICC_1,3_ was 0.983 (95% CI = 0.969–0.991). The integer values of 100%, 70%, and 40% of each individual’s maximum abdominal trunk muscle strength were rounded off and calculated and set as the target value for each training mode of RECORE^®^. Subsequently, after preparing the skin by shaving and alcohol washing to minimize skin impedance, disposable electrodes (Blue Sensor M-00-S, Medicotest, Olstykke, Denmark) were applied to the external oblique (EO), internal oblique (IO), rectus abdominis (RA), and multifidus (MF) muscles of the dominant limb side. The placement locations were as follows: The EO electrode on the inferior margin of the eighth rib, the IO electrode 2 cm medial inferior to the superior anterior iliac spine, the RA electrode approximately 3 cm lateral to the umbilicus, and the MF electrode at the level of the L5 spinous process, above the space between L1 and L2 in line from the caudal posterior iliac spine [51,52,53]. The distance between all electrodes was maintained at 2 cm. The RECORE^®^ was then worn with electrodes affixed to the trunk, and the abdominal trunk muscles were exerted to match the target value line set in the training mode of the RECORE^®^. The RECORE^®^ training mode provided visual feedback of exerted muscle strength on the LCD screen. Measurements were taken for 5 s with muscle activity, followed by a 30 s break, for a total of three measurements. The target values (100%, 70%, and 40%) measurement order was randomized by a random number table. The rest between trials was 2 min. The 3 s data during the 5 s EMG recorded in each condition were filtered with a bandpass filter with a cutoff of 50–500 Hz, full-wave-rectified, and the EMG mean amplitude was calculated. EMG normalization was performed using the maximum EMG obtained during maximal voluntary contraction (MVC) following the manual muscle test procedure, expressed as a percentage of MVC (%MVC) [54]. The average %MVC values obtained from each condition were compared and analyzed. The data collected were checked for normal distribution by the Shapiro–Wilk test, and differences in muscle activation in the three conditions were analyzed using a repeated-measures analysis of variance if normally distributed or Friedman test if not normally distributed. As a result, each muscle activity was 100%, 70%, and 40%; EO was 58.8%, 37.8%, 17.8% (*p* < 0.001); IO was 71.8%, 45.1%, and 23.8% (*p* = 0.011); RA was 54.5%, 40.0%, and 21.1% (*p* < 0.001); and MF was 13.2%, 13.0%, and 7.8% (*p* = 0.003). Significant differences were observed between each condition. Thus, we indicated that abdominal trunk muscle strength exertion in RECORE^®^ reflected trunk muscle activity.

Based on the recommendations for the grip strength assessment, the following procedures were performed [55]. Participants were placed in a standing posture with the dominant upper limb at the side of the body, elbow extended, and forearm and wrist joints in a neutral position, while holding a grip strength tester (TKK-5401, Takei Kiki Kogyo, Niigata, Japan). They practiced once or twice under submaximal conditions, and the grip width was adjusted for each participant such that the second joint of the second finger was approximately 90° (almost perpendicular). The participants were instructed not to allow the grip strength tester to touch their body or clothing. Measurements were taken three times with the dominant hand, with 1 min intervals between measurements. Participants were verbally encouraged during the test. The relationship between grip strength and force moment and muscle activity of the upper limb joints has been shown [56,57]. Thus, the maximum grip strength value was recorded as the maximum upper limb muscle strength. Data were normalized by weight.

Maximum voluntary strength of the quadriceps muscle of the dominant leg was measured using a Biodex System 3 isokinetic dynamometer (Biodex Medical Systems Inc., Shirley, NY, USA). The sampling rate was 100 Hz. This instrument demonstrated sufficient reliability and validity in the measurement of moments in both clinical and research settings [58]. The measurement leg was the one with which the participant could usually kick the ball harder and was used as the dominant leg [59]. The participants sat deep with their backs at 85°, and their chest, pelvis, and thighs were immobilized using inelastic straps to minimize body movements. The knee joint was aligned with the axis of rotation of the constant-velocity dynamometer, and the lever arm attachment was placed directly above the medial part of the ankle joint and secured with an inelastic strap. Six repetitions of knee flexion–extension were performed at an angular velocity of 90°/s in the submaximal position. After 1 min intervals, the participant was instructed to flex and extend the knee three consecutive times at an angular velocity of 60°/s to the maximum knee extension angle, starting at 110° of knee flexion [60]. Participants received verbal encouragement while performing the test. The relationship between knee extensor muscle strength and overall lower extremity strength in healthy individuals has been shown [61]. Thus, the maximum knee extension value was defined as the maximum lower limb muscle strength. Data were normalized by weight.

The participants stood with both feet directly behind the starting line and were instructed to jump as far as possible. Participants were allowed to swing their arms freely during the test. They practiced once or twice under submaximal conditions, and after sufficient rest, three trials were performed with 1 min intervals between each trial. The distance from the starting line to the heel portion of the backfoot at the landing point was measured, and the maximum value was used [62]. The data were normalized to height.

### 4.5. Statistical Analysis

Data for each item were presented as the mean ± standard deviation. Normalized values were used in all the analyses. The normality of the data distribution was determined using the Shapiro–Wilk test. For trunk muscle strength, the ICC of three measurements was calculated. Pearson’s product–rate correlation coefficient and Spearman’s rank correlation coefficient were used to examine the relationship between the trunk and upper- and lower-limb muscle strength and muscle mass of the SLJ. A multiple regression analysis was then applied using the stepwise method, with SLJ as the dependent variable; trunk and upper and lower limb strength and muscle mass as independent variables; and sex, age, and BMI as adjustment factors. Then, the standardized regression coefficient β and its 95% CI were calculated. Statistical analysis was performed using SPSS version 28.0 (IBM Corp., Armonk, NY, USA), and the significance level was set at 5%.

## 5. Conclusions

The present study suggests that the performance of horizontal jumping, such as the SLJ, should consider the strength training of the trunk and upper limbs. This may provide insights into the design of training programs to improve performance in athletic activities that require a performance similar to that of the SLJ. This study also measured the entire trunk muscle group by measuring abdominal pressure for abdominal trunk muscle strength. SLJ performance has been used as an indicator for various aspects of sport, including training. This study suggests that the abdominal trunk muscle strength measurement has the potential to become a convenient instrument for measuring athletes’ trunk muscle strength.

## Figures and Tables

**Table 1:** Participants’ characteristics and measurement results of each muscle strength, muscle mass, and SLJ (*n* = 31).

Variables			
Age (years)	20.3	±	1.4
Height (cm)	165.7	±	9.0
Weight (kg)	62.4	±	12.2
Body mass index (kg/m^2^)	22.6	±	3.4
Muscle strength to BW ratio			
Trunk (%: kPa)	29.8	±	7.6
Upper limb (%: kgf)	61.2	±	9.9
Lower limb (%: Nm)	215.8	±	36.2
Muscle mass to BW ratio			
Trunk (%: kg)	37.5	±	3.2
Upper limb (%: kg)	3.4	±	0.5
Lower limb (%: kg)	13.7	±	1.4
SLJ to BH ratio (%: cm)	117.3	±	11.9

mean ± standard deviation; SLJ, standing long jump; BW, body weight; BH, body height.

**Table 2:** Association between factors that contribute to performance during the SLJ.

Normalized Variables	*p*	β	95% CI	VIF
Trunk strength	0.006 **	0.367	0.184–0.972	1.050
Upper limb strength	<0.001 **	0.608	0.432–1.036	1.050

F-value = 21.194; ** *p* < 0.01; adjusted R^2^ = 0.574; adjusted factors = sex and age; β standardized regression coefficient; CI, confidence interval; VIF, variance inflation factor.

## Data Availability

The data presented in this study are available upon request from the corresponding author.
